# 遗传性蛋白S缺乏症18例临床表现与基因分析

**DOI:** 10.3760/cma.j.issn.0253-2727.2022.01.010

**Published:** 2022-01

**Authors:** 冬雷 张, 峰 薛, 荣凤 付, 云飞 陈, 晓帆 刘, 葳 刘, 玉娇 贾, 慧媛 李, 玉华 王, 志坚 肖, 磊 张, 仁池 杨

**Affiliations:** 中国医学科学院血液病医院（中国医学科学院血液学研究所），实验血液学国家重点实验室，国家血液系统疾病临床医学研究中心，天津 300020 State Key Laboratory of Experimental Hematology, National Clinical Research Center for Blood Diseases，Institute of Hematology & Blood Diseases Hospital, Chinese Academy of Medical Sciences & Peking Union Medical College, Tianjin 300020, China

**Keywords:** 蛋白S, PROS1基因, 血栓栓塞, 高通量测序, Protein S, PROS1 gene, Thromboembolism, High throughput sequencing

## Abstract

**目的:**

分析18例遗传性蛋白S（PS）缺乏症患者的临床表现及分子致病机制。

**方法:**

对2016年7月至2019年2月就诊于中国医学科学院血液病医院的18例PS缺乏症患者进行回顾性分析，应用凝固法测定PS活性、应用发色底物法测定蛋白C（PC）和抗凝血酶（AT）活性并进行初步诊断，使用高通量测序（HTS）筛查凝血疾病相关基因变异并进行Sanger测序验证；使用Swiss-model软件进行三维结构分析。

**结果:**

18例患者中男15例，女3例，中位年龄37（14～62）岁。均有深静脉血栓栓塞病史，PS活性为12.5～48.2 U/dl。所有患者均检出PROS1基因变异，其中5个无义突变（c.134_162del/p.Leu45*、c.847G>T/p.Glu283*、c.995_996delAT/p.Tyr332*、c.1359G>A/p.Trp453*、c.1474C>T/p.Gln492*）、2个移码突变（c.1460delG/p.Gla487Valfs*9、c.1747_1750delAATC/p.Asn583Wfs*9）和1个大片段缺失（外显子9 缺失）为首次报道。此外，1例妊娠期深静脉血栓形成女性患者的PS活性为55.2 U/dl，并且检出PROC基因c.565C>T/p.Arg189Trp突变。

**结论:**

该研究新发现的基因突变丰富了与遗传性PS缺乏症相关的PROS1基因突变谱。

## Abstract (en)

**Objective:**

To analyze the clinical manifestations and molecular pathogenesis of 18 patients with inherited protein S（PS）deficiency.

**Methods:**

Eighteen patients with inherited PS deficiency who were admitted to the Institute of Hematology & Blood Diseases Hospital from June 2016 to February 2019 were analyzed: activity of protein C（PC）and antithrombin（AT）, PS activity were measured for phenotype diagnosis; high throughput sequencing（HTS）was used for screening of coagulation disease-related genes; Sanger sequencing was used to confirm candidate variants; Swiss-model was used for three-dimensional structure analysis.

**Results:**

The PS:C of 18 patients ranged from 12.5 to 48.2 U/dL. Among them, 16 cases developed deep vein thrombosis, including 2 cases each with mesenteric vein thrombosis and cerebral infarction, and 1 case each with pulmonary embolism and deep vein thrombosis during pregnancy. A total of 16 PROS1 gene mutations were detected, and 5 nonsense mutations（c.134_162del/p.Leu45*, c.847G>T/p.Glu283*, c.995_996delAT/p.Tyr332*, c.1359G> A/p.Trp453*, c.1474C>T/p.Gln492*）, 2 frameshift mutations（c.1460delG/p.Gla487Valfs*9 and c.1747_1750delAATC/p.Asn583Wfs*9）and 1 large fragment deletion（exon9 deletion）were reported for the first time. In addition, the PS:C of the deep vein thrombosis during pregnancy case was 55.2 U/dL carrying PROC gene c.565C>T/p.Arg189Trp mutation.

**Conclusion:**

The newly discovered gene mutations enriched the PROS1 gene mutation spectrum which associated with inherited PS deficiency.

蛋白S（PS）是一种依赖维生素K的单链糖蛋白，由Discipio于1977年在美国西雅图分离成功[1]。人类PS由定位于染色体3q11.2的PROS1基因编码，跨越大约80 kb，包含15个外显子，其cDNA长度约为1.8 kb，编码的前体蛋白切除24个氨基酸残基的前导肽和17个氨基酸残基后，形成635个氨基酸组成的成熟蛋白，从N端依次为γ羧基谷氨酸（Gla）区域、凝血酶敏感区域（TSR）、4个表皮生长因子（EGF）结构域和1个性激素结合球蛋白同源结构域（SHBG），其中SHBG包括2个层黏连蛋白G（LamG）区域[2]。PS在肝脏合成后主要分布于外周血浆，大约60％的总PS与C4b补体蛋白结合，约40％以游离形式存在，后者发挥主要的活性[3]。作为重要的抗凝蛋白，一方面游离PS是活化蛋白C（APC）的辅因子，可以间接促进凝血因子Ⅴa（FⅤa）和FⅧa的水解失活[4]；另一方面PS也可以与FVa和FⅩa可逆性结合，直接抑制凝血酶原复合物的活性[5]；此外，PS还可以与FⅧa结合，从而抑制FⅩ的激活。

遗传性PS缺乏症是一种常染色体不完全显性遗传疾病，发病机制是由于PS编码基因PROS1发生功能缺失型突变，导致PS绝对数量或生物学功能受损[6]，其临床表现以静脉血栓形成（VTE）多见，部分患者也可发生多发性动脉血栓、脑梗死等。遗传性PS缺乏症在高加索VTE患者中的占比为2％～8％，在亚洲VTE患者中的占比为10.7％～17.8％[7]。迄今为止，人类基因突变数据库（human gene mutation database，HGMD）共收录450个PROS1基因变异，其中以点突变导致的错义或无义突变最为多见（150个），其次为小片段插入缺失（74个）和剪接突变（25个）。在本研究中，我们对18例遗传性PS缺乏症患者的临床表现和PROS1基因检测结果进行回顾性分析。

## 病例与方法

1. 病例：2016年7月至2019年2月期间，224例患者因动静脉血栓、肺栓塞或少见部位血栓而就诊于中国医学科学院血液病医院，其中18例患者被诊断为遗传性PS缺乏症。遗传性PS缺乏症的诊断基于以下标准[8]：患者临床表现为有症状的血栓栓塞性疾病，伴或不伴有血栓形成家族史，排除抗磷脂抗体、肝病、口服避孕药、肿瘤、糖皮质激素替代疗法和慢性炎症等因素下PS活性水平持续<60 U/dl。纳入本研究的遗传性PS缺乏症患者及家庭成员均知情同意。

2. 血栓止血实验室检测：应用Sysmex CS5100全自动凝血分析仪检测抗凝血酶（AT）、蛋白C（PC）、PS活性，其中AT和PC活性测定采用发色底物法，PS活性测定采用凝固法。检测均距患者血栓事件至少1个月，AT活性检测距患者停用肝素至少24 h，PC、PS活性检测在患者停止抗凝治疗后2周进行。

3. 基因测序：留取患者及血缘相关的部分亲属静脉血液标本3～5 ml，采用DNA提取试剂盒（北京天根生化科技有限公司产品）提取外周有核细胞基因组DNA。高通量测序检测包含PROS1在内的76个出凝血疾病相关基因，其中PROS1、PROC和SERPINC1基因的蛋白编码区域（CDS）覆盖度均为100％，且覆盖了FⅤ Leiden（FⅤ Arg506Gln）和F2基因G20210A变异。在Ion Torrent测序平台（美国赛默飞世尔科技公司产品）完成高通量测序（HTS），所有样本测序靶向区域平均测序深度均>400×，其中测序深度≥100×的读段（reads）比例>90％。测序数据与人类参考基因组hg19进行比对，采用人群数据库（1000Genomes、gnomAD、ESP6500和自建数据库等）、功能预测数据库（SIFT、PolyPhen-2和Mutation Taster等）以及疾病数据库（HGMD专业版、ClinVar和ISTH-PSD[9]数据库）进行注释。根据2015年版美国医学遗传学与基因组学学会（ACMG）指南筛选致病变异或可能致病变异，并使用Sanger测序进行验证。采用Ion AmpliSeq™custom panel测序的拷贝数变异可视化方法进行拷贝数变异分析[10]。

4. 蛋白结构预测：根据AlphaFold中的PS蛋白预测模型（AF-P07225-F1）[11]，使用Swiss-Pdb Viewer v4.0.1软件分析突变前后由氨基酸变化引起PS蛋白空间结构改变。

## 结果

1. 一般资料及临床表现：18例患者中，男15例，女3例，中位年龄37（14～62）岁，PS∶A 12.5～48.2 U/dl。截止到就诊，18例患者均有深静脉血栓病史（详见表1）。所有患者父母均无血缘关系。

**表1:** 18例遗传性蛋白S缺乏症患者一般资料及抗凝蛋白检测结果

例号	性别	年龄（岁）	临床表现	PS∶A（U/dl）	PC∶A（U/dl）	AT∶A（U/dl）	血栓病史家族成员
1	女	19	肺栓塞	36.2	107.2	113.3	无
2	男	44	深静脉血栓	20.9	92.6	96.8	父亲
3	女	41	深静脉血栓	21.2	149.8	121.2	母亲、姨母
4	男	37	深静脉血栓，脑梗死	32.4	94.0	89.2	父亲
5	男	16	肠系膜静脉血栓	14.5	98.6	115.1	无
6	男	46	深静脉血栓	26.9	89.3	106.4	父亲
7	男	51	深静脉血栓	40.2	101.6	94.7	无
8	男	29	深静脉血栓	30.0	78.8	110.0	母亲
9	男	38	深静脉血栓	22.3	85.3	99.7	父亲、叔叔
10	女	30	妊娠期深静脉血栓	20.0	55.2	87.9	无
11	男	35	深静脉血栓	19.8	90.2	98.1	无
12	男	45	深静脉血栓，肠系膜静脉血栓	48.2	93.8	128.3	无
13	男	14	深静脉血栓，肺栓塞	12.5	71.6	102.7	无
14	男	62	深静脉血栓，脑梗死	41.5	95.8	100.6	无
15	男	19	深静脉血栓	44.9	89.4	85.2	无
16	男	33	深静脉血栓，肠系膜静脉血栓	27.5	88.6	79.9	母亲
17	男	38	深静脉血栓	35.7	133.1	76.7	无
18	男	32	深静脉血栓	46.7	93.7	100.9	母亲

注：PS∶A：蛋白S活性，参考值70.0～125.0 U/dl；PC∶A：蛋白C活性，参考值70.0～140.0 U/dl；AT∶A：抗凝血酶活性，参考值75.0～125.0 U/dl

2. PROS1基因检测结果：18例患者检测到16个PROS1基因杂合突变（表2），包括7个无义突变（c.100C>T/p.Gln34*、c.134_162del/p.Leu45*、c.847G>T/p.Glu283*、c.995_996delAT/p.Tyr332*、c.1359G>A/p.Trp453*、c.1474C>T/p.Gln492*和c.1680T>A/p.Tyr560*）、3个错义突变（c.155G>A/p.Gla52Asp、c.1063C>T/p.Arg355Cys、c.1095T>G/p.Asn365Lys）、3个移码突变（c.580delT/p.Ser194Glnfs*14、c.1460delG/p.Gla487Valfs*9、c.1747_1750delAATC/p.Asn583Wfs*9）、2个剪接位点突变（c.1493-17T>C、c.1871-1G>T）以和1个大片段缺失（外显子9缺失），以上突变在整个基因中的位置见图1。此外，在例10中检测到PROC基因c.565C>T/p.Arg189Trp杂合突变。

**表2:** 18例遗传性蛋白S缺乏症患者PROS1基因检测结果

例号	突变位点（基因：核苷酸改变/氨基酸改变）	突变位置/结构域	突变类型/状态
1	PROS1:c.155G>A/p.Gla52Asp	Exon2/GLA	错义突变/杂合
2	PROS1:c.1680T>A/p.Tyr560*	Exon14/LamG2	无义突变/杂合
3	PROS1:c.1474C>T/p.Gln492*	Exon12/LamG2	无义突变/杂合
4	PROS1:c.1359G>A/p.Trp453*	Exon12/LamG1	无义突变/杂合
5	PROS1:c.1747_1750delAATC/p.Asn583Wfs*9	Exon14/LamG2	移码突变/杂合
6	PROS1:c.995_996delAT/p.Tyr332*	Exon10/LamG1	无义突变/杂合
7	PROS1:c.580delT/p.Ser194Glnfs*14	Exon6/EGF2	移码突变/杂合
8	PROS1:c.100C>T/p.Gln34*	Exon2/PRO	无义突变/杂合
9	PROS1:c.1871-1G>T/--	Intron14/Splicing	剪接突变/杂合
10	PROS1:exon9缺失/--	Exon9/LamG1	大片段缺失/杂合
	PROC:c.565C>T/p.Arg189Trp	Exon7/--	错义突变/杂合
11	PROS1:c.847G>T/p.Glu283*	Exon8/EGF4	无义突变/杂合
12	PROS1:c.1063C>T/p.Arg355Cys	Exon10/LamG1	错义突变/杂合
13	PROS1:c.1460delG/p.Gla487Valfs*9	Exon12/LamG2	移码突变/杂合
14	PROS1:c.1095T>G/p.Asn365Lys	Exon10/LamG1	错义突变/杂合
15	PROS1:c.1493-17T>C/--	Intron12/Splicing	剪接突变/杂合
16	PROS1:c.1680T>A/p.Tyr560*	Exon14/LamG2	无义突变/杂合
17	PROS1:c.134_162del/p.Leu45*	Exon2/GLA	无义突变/杂合
18	PROS1:c.1063C>T/p.Arg355Cys	Exon10/LamG1	错义突变/杂合

注：PROS1基因参考转录本为NM_000313.4，PROC基因参考转录本为NM_000312.4

**图1:**
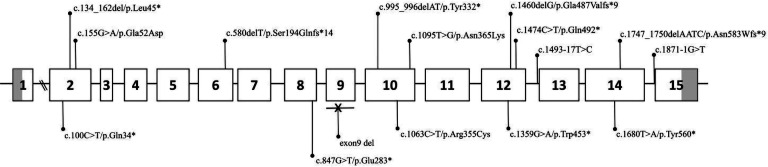
18例遗传性蛋白S缺乏症患者PROS1基因突变分布示意图 矩形框代表外显子，其中灰色部分代表非翻译区（UTR），外显子之间的横线表示内含子

3. 新发现PROS1基因突变位点分析：所有检测到的16个基因变异中5个PROS1基因变异（c.134_162del/p.Leu45*、c.847G>T/p.Glu283*、c.995_996delAT/p.Tyr332*、c.1359G>A/p.Trp453*、c.1474C>T/p.Gln492*、c.1460delG/p.Gla487Valfs*9、c.1747_1750delAATC/p.Asn583Wfs*9和外显子9缺失）为首次报道，其中c.134_162del/p.Leu45*、c.847G>T/p.Glu283*、c.995_996delAT/p.Tyr332*、c.1359G>A/p.Trp453*、c.1474C>T/p.Gln492*为无义突变，图2显示三维构象模型中这些无义突变导致PS缺失部分。c.1460delG/p.Gla487Valfs*9、c.1747_1750delAATC/p.Asn583Wfs*9为位于LamG2结构域的移码突变，均可能导致蛋白功能严重受损。此外，exon9缺失突变可能导致LamG1结构域的前38个氨基酸残基缺失，从而影响该结构域的功能。

**图2:**
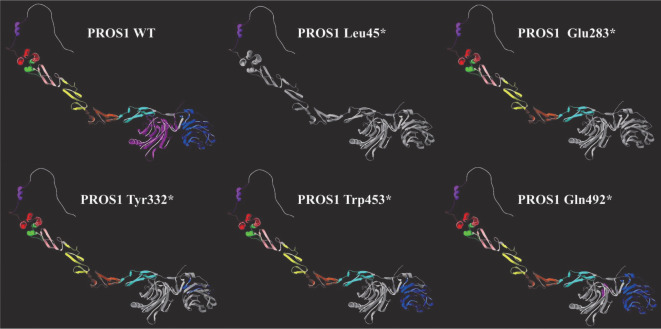
新发现PROS1基因无义突变结果分析（灰色部分代表缺失的氨基酸残基）

## 讨论

遗传性PS缺乏症是一种常染色体不完全显性遗传性疾病，由于PROS1基因突变导致PS水平降低或功能缺陷，本病与PC缺乏和抗凝血酶AT缺乏一起构成了亚洲人群中VTE的主要遗传性风险因素[12]–[13]。PS缺乏患者发生VTE的风险比普通正常人高10倍，主要表现为复发性静脉血栓形成，约有60％的患者表现为深静脉血栓，45％发生浅表静脉血栓形成，45％有肺血栓栓塞症，少数表现为脾静脉、锁骨下静脉、肠系膜静脉、前矢状窦静脉血栓形成[14]。动脉血栓形成的发生率较低，可表现为脑、冠状动脉及外周动脉血栓形成[15]。

本组PS患者中，男性占比超过83％，较小的样本量可能是导致本研究中高度性别偏倚结果的直接原因。需要指出的是，在之前的报道也发现遗传性PS缺乏症患病群体中的性别偏倚与常染色体遗传模式并不一致，生理状态下男性的基础PS水平较女性高[16]，并且男性首次发生VTE的风险是女性的3.2倍，而血栓复发的风险是女性的3.6倍[17]，因而男性可能更易因血栓形成而就诊。

在所有遗传性PS缺乏症患者中均检测到PROS1基因突变，由于本研究中先证者是在排除获得性危险因素（口服避孕药物、肝病、抗凝药物等）的干扰后，借助于出凝血实验室进行表型检查，最终诊断为PS缺乏症，因此大大提高了基因检测阳性率。先前的一些研究描述了突变分布与PS缺乏类型之间的关系[2],[6]，引起PS抗原缺乏的突变一般可以分布在整个PROS1基因区域，而引起PS单纯活性缺陷的突变通常位于Gla和EGF结构域[2]。我们在本次研究中仅检测了PS活性，而缺乏游离PS和总PS抗原水平的结果，未能对患者进行精准分型，是本项研究的局限性之一。

无义突变是本组病例PROS1基因检出最多的突变类型。一般而言，相对于其他突变类型，无义突变对PROS1基因的影响比较明确且较为重大，本研究中携带无义突变患者的PS∶A水平为19.8～36.2 U/dl，由于其他变异类型的患者数量较少，我们并未进行不同突变类型之间PS∶A水平的比较。尽管结果部分显示了无义突变导致PS蛋白截断后的剩余氨基酸残基，但在真核生物中，当提前终止密码子（PTC）距离最后1个外显子连接复合体（EJC）结合位置上游超过55个核苷酸时，mRNA质量监控机制有可能通过无义介导的mRNA降解（NMD）途径识别和降解含有PTC的转录产物，从而防止有潜在毒性的截短蛋白的产生[18]。因此，仍需要进一步从转录水平或蛋白水平研究这些突变导致疾病发生的确切机制。

本研究中发现2个已报道的PROS1基因错义突变：c.155G>A/p.Gla52Asp和c.1063C>T/p.Arg355Cys。c.155G>A/p.Gla52Asp位于Gla结构域，对于PS-磷脂之间高亲和力相互作用发挥重要功能，体外研究显示PROS1 Gla52Asp重组蛋白与针对野生型PS蛋白Gla结构域特异性抗体以及阴离子磷脂囊泡的亲和力都显著降低，并且在体外灭活因子Ⅴa的活性较野生型PS蛋白减弱15.2倍[19]。PROS1 p.Arg355Cys位于SHBG结构域中LamG1的第一个球状结构域，是PS缺乏症患者较为常见的致病变异[20]–[21]。

剪接位点变异通常会破坏原有的剪接方式导致外显子跳跃，c.1871-1G>T位于14号内含子与15号外显子交界的经典剪接位点，该突变可引起mRNA的异常剪接，改变翻译阅读框并且提前终止翻译过程，最终产生不稳定的PS突变蛋白[22]。在极为罕见的情况下内含子的突变会激活隐秘的剪接位点，位于12号内含子的c.1493-17T>C突变较为临近内含子剪接受体位点的-AG-双碱基对，因此可能通过干扰野生型转录产物的正常剪接过程影响PS水平[8]。

HTS普遍适用于以点突变或小片段缺失插入为主要变异形式的孟德尔单基因遗传病的基因检测，然而随着生物信息学分析手段的进步，使得大片段缺失等拷贝数变异（CNV）的检测成为可能。大片段的缺失或重复在遗传性PS缺乏症患者中并不少见，本研究中发现了1例外显子9整体缺失的妊娠期血栓形成病例，该患者除了PS活性水平降低，同时也携带有亚洲PC缺乏症人群最常见的PROC基因c.565C>T/p.Arg189Trp突变。PC和PS联合缺陷很少见，但血栓形成风险显著增加[23]–[24]，该患者在PS和PC两种抗凝蛋白存在缺陷的情况下又叠加了妊娠状态，最终表现为VTE。

综上所述，本研究报道了18个遗传性PS缺乏症病例的临床表现及PROS1基因突变检测结果，丰富了遗传性PS缺乏症的基因突变谱，有助于该病遗传咨询和产前诊断的开展。新发现基因突变的致病分子机制尚需继续研究。
